# A parallel genome-wide RNAi screening strategy to identify host proteins important for entry of Marburg virus and H5N1 influenza virus

**DOI:** 10.1186/s12985-015-0420-3

**Published:** 2015-11-24

**Authors:** Han Cheng, Katie Koning, Aileen O’Hearn, Minxiu Wang, Emily Rumschlag-Booms, Elizabeth Varhegyi, Lijun Rong

**Affiliations:** Department of Microbiology and Immunology, College of Medicine, University of Illinois at Chicago, Chicago, IL 60612 USA; Present address: US Army Medical Research Institute of Infectious Diseases, Fort Detrick, MD21702 USA; Present address: Malcolm X College, Chicago, IL 60612 USA; Present address: Northeastern Illinois University, Chicago, IL60625 USA

## Abstract

**Background:**

Genome-wide RNAi screening has been widely used to identify host proteins involved in replication and infection of different viruses, and numerous host factors are implicated in the replication cycles of these viruses, demonstrating the power of this approach. However, discrepancies on target identification of the same viruses by different groups suggest that high throughput RNAi screening strategies need to be carefully designed, developed and optimized prior to the large scale screening.

**Methods:**

Two genome-wide RNAi screens were performed in parallel against the entry of pseudotyped Marburg viruses and avian influenza virus H5N1 utilizing an HIV-1 based surrogate system, to identify host factors which are important for virus entry. A comparative analysis approach was employed in data analysis, which alleviated systematic positional effects and reduced the false positive number of virus-specific hits.

**Results:**

The parallel nature of the strategy allows us to easily identify the host factors for a specific virus with a greatly reduced number of false positives in the initial screen, which is one of the major problems with high throughput screening. The power of this strategy is illustrated by a genome-wide RNAi screen for identifying the host factors important for Marburg virus and/or avian influenza virus H5N1 as described in this study.

**Conclusions:**

This strategy is particularly useful for highly pathogenic viruses since pseudotyping allows us to perform high throughput screens in the biosafety level 2 (BSL-2) containment instead of the BSL-3 or BSL-4 for the infectious viruses, with alleviated safety concerns. The screening strategy together with the unique comparative analysis approach makes the data more suitable for hit selection and enables us to identify virus-specific hits with a much lower false positive rate.

## Background

Emerging and re-emerging human viral pathogens pose one of the major public health concerns since effective countermeasures are not available to detect, prevent, and treat these viral diseases [1]. The 2013–2015 West Africa Ebola epidemic, with more than 25,000 people infected and more than 12,000 deaths, underlines the global challenge dealing with the infection and diseases associated with these viruses. To develop prophylactic and therapeutic options, it is important to understand how these viruses interact with their hosts. Therefore efforts have been made to identify and characterize host factors which are involved in viral replication and infection for different viruses. A recently developed technology, referred to as genome-wide RNA interference (RNAi) based screening [2], has been employed by different groups to identify host factors systematically, and a large number of host factors have been implicated as critical for infection for viruses such as human immunodeficiency virus-1 (HIV-1 [3–5]) and influenza H1N1 virus [6–9], providing mechanistic insights on the virus/host interactions. However, the hit overlaps between screens by different groups are pretty low, with 3–6 % for HIV-1 screens and 1–12 % for influenza virus screens [10], raising concerns about the potential high false positive rates and even some skepticism on the utility of this technology. Thus, to avoid potential screening artifacts and other issues, it is prudent to carefully design high throughput RNAi screening strategies and to optimize the parameters in the pilot experiments prior to the large-scale screens.

Another obstacle in working with highly pathogenic viruses is that they require high containment facilities (biosafety level-3 or 4, or BSL-3 or BSL-4), which are not readily available for many researchers. However this problem can be often circumvented by an HIV-1 based surrogate system in the entry studies and in the drug discovery efforts to identify and develop antivirals targeting the entry process [11–17]. This HIV-1 based surrogate assay is particularly amenable for high throughput screens because it is safe and robust. In this report, we describe a genome-wide RNAi high throughput screen strategy, referred to as parallel genome-wide RNAi screen, which allows us to quickly identify host factors important for the entry process of highly pathogenic viruses. This strategy was used for an RNAi screen to identify host proteins specific for the entry process of Marburg virus (MARV) or avian influenza virus H5N1 (AIV), demonstrating the utility of this approach.

## Results

### Development of a parallel genome-wide RNAi screen protocol

To perform a high throughput RNAi screen to identify the host factors involved in viral entry of highly pathogenic Marburg virus and avian influenza virus H5N1, which require BSL-3 and BSL-4 facilities, respectively, we adopted a surrogate system which allows us to perform the initial screening in a BSL-2 facility [18]. This human immunodeficiency virus-1 (HIV-1) based surrogate assay has been widely used by us and others to investigate the entry mechanisms of highly pathogenic enveloped viruses such as filoviruses [11, 12, 16] (Ebola and Marburg viruses), avian influenza virus H5N1 [13], and severe acute respiratory syndrome coronavirus (SARS-CoV) [19]. It has also been used to identify and develop entry inhibitors as antivirals [14, 15].

In this study, avian influenza virus H5N1 pseudovirions (AIV [13]) and Marburg virus pseudovirions (MARV [20]) were generated and used in the genome wide RNAi screen, as described in the Materials and Methods. The basic principle of this surrogate assay is based on the following two aspects: (1) AIV and MARV viral envelope glycoproteins (HA and GP, respectively) are necessary and sufficient to mediate virus entry, and (2) these glycoproteins can be efficiently incorporated into HIV-1 viral particles. Therefore these pseudovirions retain the entry property dictated by avian influenza virus H5N1 glycoprotein (HA) or Marburg glycoprotein (GP). Thus, aspects of these entry mechanisms can be evaluated despite of the surrogate nature of the viral particles. Furthermore, the pseudovirions carry a luciferase reporter gene which can be used to measure the entry activity of AIV and MARV [18].

**Fig. 1:**
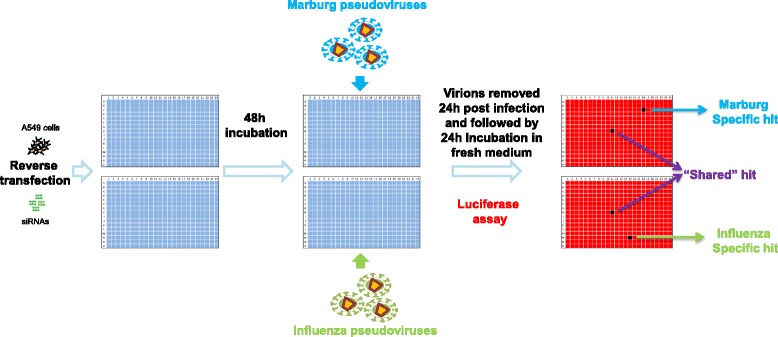
Experimental design of the parallel genome-wide RNAi screen. A549 cells are reverse transfected with siRNAs in two 384-well plates in parallel. After 48 h incubation, cells in one plate are challenged by Marburg pseudovirions and the cells in the other plate are challenged by influenza H5N1 pseudovirions. The virions are removed 24 h later and the cells are incubated with fresh medium for an additional 24 h. Virus infection is then quantified by a luciferase activity assay. siRNA only showing low signal (*black*) in an assay plate of one virus is regarded as a virus specific hit; siRNA showing low signals in assay plates of both viruses is regarded as a “shared” hit by the two viruses

The overall RNAi screening protocol is outlined in Fig. 1. The key feature of the protocol is that AIV and MARV pseudovirions were used in parallel in the RNAi screen which allowed us to reduce the number of false positives and to quickly identify Marburg-specific and flu-specific host factors (which will be further discussed below). Briefly, human A549 cells (~1000 cells/well) were reverse transfected with 10nM small interfering RNAs (siRNAs) in two identical 384-well plates simultaneously. These plates were incubated for 48 h for target gene expression knockdown, followed by challenging one plate with AIV pseudovirions and the other plate with MARV pseudovirions. Virions were replaced with fresh medium 24 h post infection and the plates were incubated for additional 24 h. Luciferase assays were then performed to quantify virus infection.

### Quality control of the screen

Screen data quality is crucial for subsequent data analysis and hit selection. Quality control of the screen is commonly indicated by the Z’ factor [21] which assesses the separation between measured signals of the positive and negative controls in an assay plate.

**Fig. 2:**
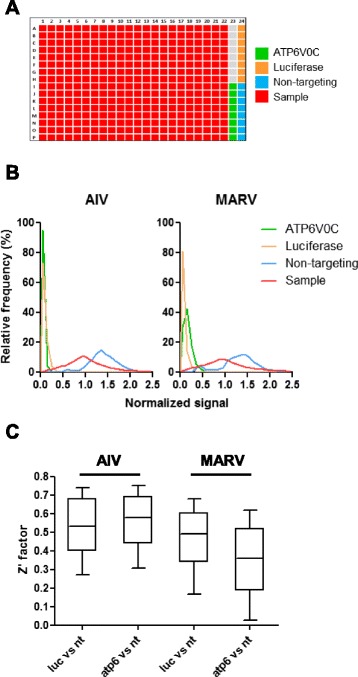
Quality control of the screen. **a** Screen plate design. A representative 384-well plate is shown to illustrate the locations of sample and control siRNAs. Sample siRNAs (*red*) are arranged in column 1 through column 22. Non-targeting siRNAs (*blue*) and siRNAs targeting ATP6V0C (*green*) or luciferase (*yellow*) are arranged in columns 23 and 24 respectively. **b** Signal distribution of samples and controls. The sample and control luciferase signals are normalized by the median signal value of all the samples in each 384-well assay plate. The normalized signal distributions in both Marburg virus (MARV) and influenza H5N1 virus (AIV) plates are plotted for sample (*red*) and controls: ATP6V0C (*green*), luciferase (*yellow*) and non-targeting (blue). **c** Z’ factors. Z’ factors are computed either by the normalized signal values of luciferase and non-targeting controls (luc vs nt) or those of ATP6V0C and non-targeting controls (atp6 vs nt). These Z’ factors are plotted for both MARV and AIV plates

In our screens, we included three controls: non-targeting siRNA as the negative control and siRNAs targeting luciferase or ATP6V0C as the positive controls. The pseudovirion carries a luciferase reporter gene, and AIV and MARV virus entry is dependent on the low-pH environment in the endosome/lysosome which is regulated by vacuolar ATPase. So siRNA targeting luciferase or ATP6V0C (a component of vacuolar ATPase) is expected to significantly reduce the final luciferase activity level. In addition, these two positive controls can serve as transfection efficiency controls, monitoring siRNA transfection. Eight control siRNAs of each type were arranged on the column 23 or 24 of a 384-well assay plate, as shown in Fig. 2a.

Figure 2b represents the normalized signal distributions of the samples and the controls. The normalized signal was obtained by dividing the measured signal in each well by the median luciferase signal of all sample wells in a plate. It is clear that both siRNAs targeting luciferase and ATP6V0C greatly reduced luciferase signal level with little variance, confirming their efficacy and excellent siRNA transfection efficiency in the screen. The samples show a broad bell-shaped signal distribution, with a little positively skewed. The signal distribution of the non-targeting control was expected to overlap with that of samples. However in our screen, this distribution was shifted to the right, showing a higher signal level. The skewed sample distribution and the shift of negative control distribution suggest that the assay plates were affected by edge effects or the positional effects (see below).

Despite of the adverse impact of the positional effects, the Z’s calculated from two control combinations (luciferase versus non-targeting and ATP6V0C versus non-targeting), are very close to 0.5, an indication of excellent assay quality (Fig. 2c). In comparison with Z’s from AIV screen plates, Z’s from MARV screen plates exhibited less favorable values and wider ranges, suggesting a larger variation in MARV screen data. This is consistent with what is shown in Fig. 2b, that is that both samples and non-targeting controls from MARV screen plates have flatter signal distributions, as compared with those from AIV screen plates.

### Alleviation of positional effects by comparative data analysis

In the aforementioned screen strategy, we were more interested in identifying virus-specific host proteins which could be revealed by a comparative analysis approach, due to the parallel nature of the screen. In this approach, the infection rates for AIV and MARV for each siRNA were obtained respectively by normalizing the measured luciferase signals with corresponding plate signal medians; the relative infection index, namely the logarithm of the ratio of their infection rates to base 2 - log_2_(ratio_MARV/AIV_), was then calculated for each siRNA to indicate specificity. Theoretically, an siRNA with relative infection index equaling to zero means no bias of the siRNA on the infections of both viruses; an siRNA inhibiting MARV infection or enhancing AIV infection results in a minus value, and an siRNA with an the opposite effect results in a positive value.

**Fig. 3:**
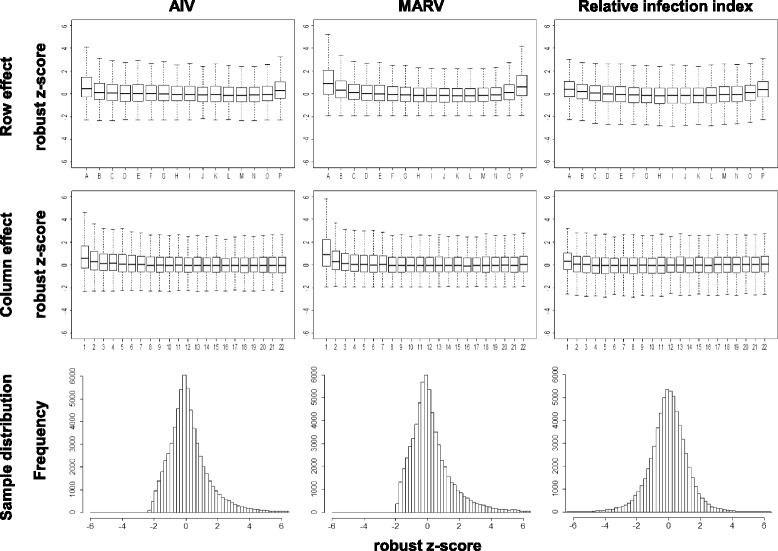
Comparative analysis reduces systematic positional effects. The normalized signals from Marburg virus (MARV) or influenza H5N1 virus (AIV) plates are used to calculate the median and median absolute deviation (MAD) respectively. Relative infection index is first calculated by using normalized signals from both virus plates and then it is used to compute the median and the MAD. The robust z-score for normalized signal or the relative infection index is then computed for each well and the robust z-score distributions across all the rows or the columns or the plates are plotted accordingly for AIV and MARV plates

We used this comparative analysis as a means to correct the observed positional effects. The robust z-score, a robust version of z-score which indicates the number of standard deviations from the mean, was employed to represent data variance. The robust z-score distributions of the normalized signals for the columns and rows from both AIV and MARV screen plates are shown in Fig. 3. Consistent with what we show in Fig. 2b, the distributions from the columns and rows are skewed towards the high z-score value area, with the outer two columns and rows more severely affected. When the relative infection index was introduced, the excess variations of the peripheral positions were greatly reduced and the distributions of columns and rows were corrected to be more symmetrical. The same is true regarding the distributions of the all siRNAs. As shown in Fig. 3 (the bottom panel), the robust z-scores of the normalized signals from both screens are heavily skewed to the right, with almost no siRNAs showing robust z-score less than −2, a recommended criterion for picking inhibitory hits. The robust z-scores of the relative infection index, however, are evenly distributed, reducing the skewed distribution caused by the positional effects and making the data more suitable for the hit selection.

### Hit selection

**Fig. 4:**
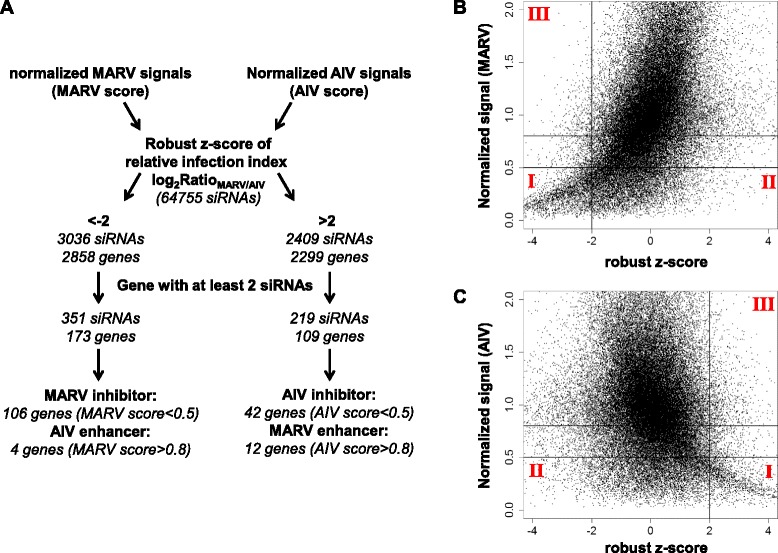
Hit selection. **a** Flowchart of hit selection process. (**b** and **c**) Enrichment of hits. For each siRNA, the normalized signal from Marburg virus (MARV) or influenza H5N1 virus (AIV) plates is plotted against the robust z-score of the relative infection index. Hits in area I are classified as specific inhibitory hits against either MARV or AIV entry. Hits in area II are classified as non-specific inhibitory hits against either MARV or AIV entry. Hits in area III are classified as hits that enhance virus entry of either MARV or AIV

Since the relative infection index approach can reduce the systematic errors, we combined it with the individual virus infection rates to identify virus-specific host factors. As presented in Fig. 4a, the hit selection began with normalizing the measured luciferase signals by the plate signal medians for MARV and AIV, respectively. These normalized scores were used for calculating the relative infection index, from which a robust z-score was then computed for each siRNA. Thresholds of ±2 were adopted for filtering the hits. From the total 64,775 individual siRNAs, 3,036 siRNAs targeting 2,858 genes had robust z-scores less than −2, while 2,409 siRNAs targeting 2299 genes had robust z-scores greater than 2. Another criterion (2 siRNAs per gene rule) that a gene must have at least 2 siRNAs showing similar effects was applied to further filter the hits, with the aim of reducing false positive numbers. Accordingly, 173 genes with 351 siRNAs for MARV and 109 genes with 219 siRNAs for AIV were selected, respectively. The individual virus infection rates (the normalized signals) were then employed as a reference to distinguish an inhibitory hit from the one that enhances virus infection. The robust z-scores are plotted against normalized signals of each virus in Fig. 4b and c. A robust z-score less than −2 in Fig. 4b means that the MARV infection rate is much less than AIV infection rate for the corresponding siRNA. This may be a result of inhibited MARV infection or of enhanced AIV infection. Thus a MARV score (normalized signal) below 0.5 was used to indicate a MARV inhibitory hit, which is represented in area I in Fig. 4b. A gene with at least two siRNAs’ MARV scores < 0.5 was used to filter the hits in area I, with 106 genes being identified as host factors that inhibit MARV entry. On the contrary, MARV scores greater than 0.8, which means the siRNA has little effect on inhibiting MARV infection, were used to indicate a hit enhancing AIV entry. Area III in Fig. 4b represents these hits and 4 host factors were identified with the 2 siRNAs per gene rule. Similarly, area I in Fig. 4c represents AIV inhibitory hits and 42 genes were identified; area III represents the hits enhancing MARV entry with 12 genes finally identified.

The use of relative infection index together with individual virus infection rate and the 2 siRNAs per gene rule is a powerful means to identify virus-specific host factors and to reduce the false positive rate. The parallel nature of the screen strategy makes the results from the two viruses highly comparable and the relative infection index obtained by comparison easily establishes virus-specificity. As shown in Fig. 4b and c, only hits in areas I and III were picked for further analysis. Though many other siRNAs also showed inhibitory effects (area II), these siRNAs were ruled out for lack of virus-specificity, greatly reducing the false positive rate. Further, the 2 siRNAs per gene rule helped to reduce false positive which may result from off-target effects or systematic errors.

A selected short list of EBOV or AIV entry-related genes is shown in Table 1 to demonstrate the power of the parallel screen strategy described in this report. Four of seven subunits of the coatomer 1 (COP-I) vesicular transport complex (i.e., ARCN1, COPA, COPB2, and COPG) were reported to be critical for influenza virus replication by others [8], but only ARCN1 was identified as an AIV-specific hit in this screen (Table 1). The other three proteins were identified in our screen as “shared” host factors for both AIV and MARV (data not shown). Proton-transporting V-type ATPase was implicated as the host factor for influenza virus infection by the previous screens [8], and we show that only two subunits of ATPase, APT6V0C and ATP6AP2, are AIV-specific. A number of other subunits (i.e. ATP6AP1, ATP6V0B, ATP6V0D1, ATP6V1A and ATP6V1B2) are not specific to AIV but are “shared” host factors by both viruses. As for MARV, a few essential MARV-specific host factors were identified by the screen in this study. Cathepsin L (CTSL), which primes the filovirus glycoprotein in the endosome [22], EXT1 [23], which is involved in biosynthesis of heparan sulfate, and Niemann-Pick disease, type C1 (NPC1 [24, 25]), showed strong bias against MARV entry (Table 1). Also, HOPS complex, which mediates fusion of the endosome and lysosome, was identified as a host factor for Ebola virus infection in a gene-trap based screen [24], and we identified VPS 16, a subunit of HOPS complex, as a MARV-specific hit in the current screen.

**Table 1:** A selected virus entry gene list from the parallel genome-wide RNAi screen

		siRNA #1		siRNA #2		siRNA #3	
Gene	Virus	score^a^	rzscore^b^	score	rzscore	score	rzscore
NPC1	MARV	0.32	−3.25	0.29	−3.35	0.22	−3.73
	AIV	1.37		1.28		1.16	
EXT1	MARV	0.22	−3.48	0.36	−2.98	0.87	−0.07
	AIV	1.03		1.35		0.89	
VPS16	MARV	0.26	−1.74	0.36	−2.31	0.11	−4.93
	AIV	0.57		1.01		0.96	
CTSL	MARV	0.90	1.09	0.38	−1.82	0.11	−3.92
	AIV	0.55		0.86		0.61	
TIM1	MARV	2.06	1.21	1.33	−0.2	1.75	0.44
	AIV	1.18		1.44		1.42	
FOLR1	MARV	1.68	−0.33	0.57	0.46	0.45	0.36
	AIV	1.93		0.46		0.38	
ARCN1	MARV	1.17	4.71	0.03	−6.62	0.22	3.35
	AIV	0.14		0.57		0.05	
ATP6V0C	MARV	0.27	1.32	0.11	2.03	0.26	3.53
	AIV	0.15		0.04		0.05	
ATP6V0D1	MARV	0.08	1.05	0.23	−1.59	0.04	3.93
	AIV	0.05		0.47		0.23	
ATP6AP1	MARV	0.16	0.80	0.57	−1.14	0.8	6.31
	AIV	0.11		0.95		0.05	

For each gene, three siRNAs are shown with their robust z-score of relative infection index. siRNAs with positive values are putative influenza H5N1 virus specific host factors; siRNAs with negative values are putative Marburg virus specific host factors

^a^score is normalized luciferase signal by plate median signal

^b^rzscore is the robust zscore of relative infection index (log_2_Ratio_MARV/AIV_)

It is interesting to note that two host proteins, FOLR1 [26] and TIM1 [27], which were previously reported as host factors important for filovirus entry, were not identified as the hits in this study (Table 1). The results from this report are consistent with a study which showed that FOLR1 was not critical for filoviral entry [28]. However, more studies are needed to evaluate the potential role of TIM1 in filoviral entry.

## Discussion

This report describes a new strategy, referred to as a parallel genome-wide RNAi screen, to identify host factors which are important for entry of enveloped viruses. This strategy is particularly useful for highly pathogenic viruses such as Ebola/Marburg viruses and avian influenza virus H5N1 (or bird flu) since the HIV-1 based surrogate system allows us to perform high throughput screens in the biosafety level 2 (BSL-2) containment instead of the BSL-4 or BSL-3 for the infectious viruses with alleviated safety concerns. More importantly, the parallel nature of the strategy allows us to easily identify the host factors for a specific virus with a greatly reduced number of false positives in the initial screen, which is one of the major problems with high throughput screening. The power of this strategy is well illustrated by a genome-wide RNAi screen for identifying the host factors important for MARV and/or AIV as described in this study.

Genome-wide RNAi screening has been widely used to identify host proteins involved in replication and infection of different viruses, and numerous host factors are implicated in the replication cycles of these viruses, demonstrating the power of this approach. However, it is clear that these RNAi screens, even performed on the same virus by different groups, do not always identify the same set of host proteins, suggesting that high throughput RNAi screening strategies need to be carefully developed and optimized prior to the actual screening. Thus several features of the screening strategy described here are attractive to identify host proteins involved in viral entry of highly pathogenic enveloped viruses. One obvious advantage of this strategy is that it can distinguish virus-specific hits from the “shared” hits. Because each virus in the screen also serves as a control for the other virus, an siRNA showing effects on one virus but not on the other one will be tentatively classified as a specific hit, as illustrated in Fig. 1. On the other hand, if an siRNA shows similar inhibitory effects on both viruses, the scenarios are more complicated: (1) the siRNA may be toxic to the cells; (2) it may induce an off-target effect; (3) it may target an HIV-1 related process after the pseudovirion is released from the endosome/lysosome; (4) it may target the shared host factors by both viruses (i.e. AIV and MARV in this study).

Another merit of the current strategy is that it can reduce the false positive rate. The reasons for high false positive rates in a genome-wide RNAi screen are due to a lack of replicates and siRNA off-target effects. In this study, each siRNA was actually tested twice, but with two different viruses. For an siRNA specific to one virus entry, the possibility of obtaining similar results for two different viruses is pretty low, greatly decreasing the false positive rate. For shared hits, the false positive rate also decreases owing to the duplicate nature of this strategy. Further, an siRNA hit due to an off-target effect is likely to affect both viruses' infection and can thus be classified as a “shared” hit, resulting in a lower false positive rate for the virus-specific hit. In addition, it has been recently shown that virus infection rate is largely determined by the population context (e.g. local cell density) of the target cell which can be affected by gene perturbations through RNAi [29]. Because the screens in this study were performed in parallel, the cell population context was, to a large extent, the same for the two viruses, making the results from the two screens highly comparable.

We have observed the position effects in this study, which are a commonly observed phenomenon in RNAi screens, as a result of the long incubation period needed for RNAi assays. Thus it can bring more noise to the screen and results in distortions of the true effects of siRNAs. A number of pre and post-screening correction methods have been developed to battle these effects. It has been suggested that the controls evenly scattered over an assay plate by careful plate design can be used to adjust the systematic errors [30]. However, most commercial available siRNA libraries only have the peripheral wells reserved for controls; in our case, only the outer 23 and 24 columns are available for controls, leaving little room for control-based systematic error adjustment. Also, a few mathematical algorithms have been proposed to reduce the effects [31]. These methods usually assume a large difference between a positive and a negative response and the sparseness of positive responses, which is not true for RNAi screens. In fact, RNAi is a very complicated biological phenomenon; even an siRNA targeting an unrelated gene may induce somewhat positive phenotype. Thus modifying the original screen data with additional correction factors by those proposed mathematical treatment may lead to more man-made artifacts.

In our comparative analysis approach, however, we are able to alleviate the positional effects without introducing a correction factor. The positional effects are largely due to the different evaporation rates across the plate which leads to a multiplicative bias to the measured signal. Because the screens were performed in parallel, the duplicate wells with a same relative position in two plates were screened simultaneously, making them subject to a similar positional effect. This multiplicative bias can thus be corrected by simply dividing virus infection rate by the rate of the other one. In our case, the relative infection index was used to reduce the bias and the results we presented in Fig. 3 have clearly validated this approach.

It should be pointed out that this report describes a powerful high throughput screening strategy which allows the initial identification of host proteins which may play a role in AIV- or MARV-specific entry. However, the role of each putative host factor has to be individually confirmed and validated, and one of the challenges is to prioritize and select the host factors to be carefully characterized.

## Conclusions

We have developed a parallel genome-wide RNAi screen strategy to identify specific host factors to either AIV or MARV entry. Implementation of this strategy generated two sets of data and a comparative analysis approach is proposed. Our screening strategy together with the unique comparative analysis approach alleviates the systematic positional effects, makes the data more suitable for hit selection, and enables us to pick virus-specific hits with a much lower false positive rate. This strategy, we believe, can be easily adapted to other screens with the aim of increasing screen specificity and reducing false positive rates.

## Methods

### Cell culture and plasmids

Human 293 T embryonic kidney cells and human lung epithelial A549cells were cultured in Dulbecco's modified Eagle's medium (DMEM, Cellgro) supplemented with 10 % fetal bovine serum (FBS, Gibco), 100 μg/mL of streptomycin and 100 units of penicillin (Invitrogen). The avian influenza virus H5N1/HIV pseudovirions (AIV) and Marburg virus/HIV pseudovirions (MARV) were generated from the following plasmids: hemagglutinin (HA), isolated from a highly pathogenic avian influenza virus, A/Goose/Qinghai/59/05 (H5N1) [13]; neuraminidase (NA) from A/Puerto Rico/8/1934 (H1N1) [13]; Marburg virus glycoprotein GP (MGP) [20]. The HIV-1 proviral vector pNL4-3.Luc.R^−^E^−^ [32, 33] was obtained through the NIH AIDS Research and Reference Reagent program.

### Production of pseudovirions

AIV and MARV pseudovirions were produced by transient co-transfection of human 293 T cells using a polyethylenimine (PEI)-based transfection protocol [11]. Replication-defective HIV vector (pNL4-3.Luc.R^−^E^−^) together with plasmids encoding MGP or HA/NA were used for transient co-transfection into 293 T producer cells. Six hours after transfection, cells were washed with phosphate-buffered saline (PBS), and 40 mL of fresh medium was added to each plate (150 mm). Forty-eight hours post transfection, the supernatants were collected and filtered through 0.45 μm pore size filter (Nalgene). The pseudovirion stocks were stored at 4 °C prior to use.

### siRNA libraries and controls

Three siRNA libraries (Silencer® Select Human Druggable Genome siRNA Library V4, Human Druggable Genome V4 Extension and Human Genome V4 siRNA Extension) were purchased from Ambion (Austin, TX). These libraries contain 64,755 siRNAs targeting 21,585 human genes (each gene has three distinct siRNAs). Daughter plates were prepared in 384-well format at a 250nM concentration in water. Controls include siRNA targeting ATP6V0C or luciferase and non-targeting siRNA. All control siRNAs were purchased from Ambion.

### Genome-wide RNAi screening

All siRNAs were arrayed in 384-well plates. For each assay plate well, 2 μl siRNA was mixed with 0.1 μl Lipofectamine RNAiMAX (Invitrogen) and 9.9 μl Opti-MEM (Invitrogen). After 20 min incubation at room temperature, 38 μl cell suspension of 1000 A549 cells was added, resulting in a final siRNA concentration of 10nM. Cells were incubated at 37 °C and 5 % CO_2_ for 48 h. Then the medium was removed and the cells were challenged with 30 μl MARV or AIV virions in parallel. Virions were removed 24 h later and 30 μl fresh DMEM supplemented with FBS and antibiotics was added to each well. After another 24 h incubation, 15 μl of neolite luciferase substrate (PerkinElmer, Waltham, MA) was mixed in, incubated for 5 min, and luciferase activity was measured with an Envision plate reader (PerkinElmer). All multi-well pipetting procedures were conducted by the JANUS automated liquid handling system (PerkinElmer).

### Data analysis

The measured luminescence signal was analyzed using the statistical programming language R. The signal in each well was first normalized by the median value of all the samples from the same plate.

The Z’ factor [21] was calculated from the normalized signals of not-targeting and ATP6V0C controls or from those of not-targeting and luciferase controls on each plate with the following equation: Z ' = 1 − 3(Std_high ‐ signal_ + Std_low ‐ signal_)/(Mean_high ‐ signal_ − Mean_low ‐ signal_).

For each siRNA, the relative infection index was calculated from the corresponding normalized signals from the MARV and AIV plates with the following equation: relative infection index = log_2_ (normalized signal_MARV_/normalized signal_AIV_).

Robust z-score was calculated for both normalized signal and relative infection index. First, median absolute deviation (MAD [34]) was calculated as follows:

$$ \mathrm{MAD}=1.4826\times \mathrm{median}\left(\left|{\mathrm{x}}_{\mathrm{ij}}-\mathrm{median}\left(\mathrm{x}\right)\right|\right), $$

and then the robust z-score was calculated for each siRNA as follows:

$$ \mathrm{robust}\ \mathrm{z}\hbox{-} \mathrm{score}=\left({\mathrm{x}}_{\mathrm{ij}}\hbox{-} \mathrm{median}\left(\mathrm{x}\right)\right)/\mathrm{MAD}, $$

where x represents the set of all the normalized signals or the relative infection indexes of the samples and x_ij_ indicates the value for a particular well at row i and column j. The constant 1.4826 is used to make MAD comparable to standard deviation.
